# 

**Published:** 1883-01

**Authors:** 


### CONTENTS.

***MISCELLANEOUS.*** PAGE

Seek the Simillimum. Carroll Dunham, . . . . . . . . . 9
Typhoid Fever: The First Prescription. Dr. Wells, . . . . . 10
New Discoveries. Ad. Lippe, M. D., Phila., . . . . . . . 12
Overtaxed Brains. R. R. Gregg, M. D., Buffalo, . . . . . . 18
Notes on "After-pains" (extract), . . . . . . . . . . 25
Rest as a Cause of Aggravation. Dr. C. Von Bœnninghausen, . . 26
Notes on Genito-urinary Therapeutics. E. J. L., . . . . . . 29
Dr. Wells' Essays on Dysentery, Diarrhœa, Typhoid Fever, etc., . . 31
A Curious Mistake, . . . . . . . . . . . . . . 31
Fiat Justitia, etc., . . . . . . . . . . . . . . 31
Circular from Bureau on Legislation, etc., American Institute. J. C. Morgan, M. D., . . . . . . . . . . . . . 32
Obituary. William A. Hawley, . . . . . . . . . . 34

***CLINICAL BUREAU.***

A Case of Gonorrhœa, with some Nuts for the Isopaths to Crack. E. W. Berridge, M. D., London, . . . . . . . . . . . 35
Solis Ictus; a Case. J. W. Thomson, M. D., Springfield, Mass., . . . 38
Hamam. in Hæmorrhoids. H. D. Baldwin, M. D., Syracuse, . . . 39

***BOOK NOTICES.***

Burnett: Essays. Bœricke & Tafel, . . . . . . . . . 40

Guernsey: Plain Talks on Avoided Subjects. M. A. Davis, . . . 40

Clapp: Visiting List. Otis Clapp & Sons, . . . . . . . . 40

Walker: Birth-time, etc., Reprint, . . . . . . . . . . 40

***APPENDIX.***

Dysentery, . . . . . . . . . . . . . . . . . 1–8

Cough Symptoms, . . . . . . . . . . . . . . . 45–50

